# The epidemiology and healthcare costs of pregnancy-related listeriosis in British Columbia, Canada, 2005–2014

**DOI:** 10.1017/S0950268824001821

**Published:** 2024-12-18

**Authors:** Antonela Ilic, Dimitra Panagiotoglou, Eleni Galanis, Marsha Taylor, Zahid A. Butt, Shannon E. Majowicz

**Affiliations:** 1 School of Public Health Sciences, University of Waterloo, Waterloo, Canada; 2 Department of Epidemiology, Biostatistics and Occupational Health, McGill University, Montreal, Canada; 3 School of Population and Public Health, University of British Columbia, Vancouver, Canada; 4 British Columbia Centre for Disease Control, Vancouver, Canada

**Keywords:** Listeriosis, *Listeria*, pregnancy, neonatal listeriosis, epidemiology, foodborne

## Abstract

This study investigated cases of pregnancy-related listeriosis in British Columbia (BC), Canada, from 2005 to 2014. We described all diagnosed cases in pregnant women (*n* = 15) and neonates (*n* = 7), estimated the excess healthcare costs associated with listeriosis, and calculated the fraction of stillbirths attributable to listeriosis, and mask cell sizes 1–5 due to data requirements. Pregnant women had a median gestational age of 31 weeks at listeriosis onset (range: 20–39) and on average delivered at a median of 37 weeks gestation (range: 20–40). Neonates experienced complications but no fatalities. Stillbirths occurred in 1–5 of 15 pregnant women with listeriosis, and very few (0.05–0.24%) of the 2,088 stillbirths in BC in the 10 years were attributed to listeriosis (exact numbers masked). Pregnant women and neonates with listeriosis had significantly more hospital visits, days in the hospital and physician visits than those without listeriosis. Pregnant women with listeriosis had 2.59 times higher mean total healthcare costs during their pregnancy, and neonates with listeriosis had 9.85 times higher mean total healthcare costs during their neonatal period, adjusting for various factors. Despite small case numbers and no reported deaths, these results highlight the substantial additional health service use and costs associated with individual cases of pregnancy-related listeriosis in BC.

## Introduction

Listeriosis, caused by the pathogen *Listeria monocytogenes*, is a significant foodborne infection, in part due to its high case fatality rate [1, 2]. Globally, listeriosis causes thousands of illnesses and deaths, and in 2010 resulted in 172,823 disability-adjusted life-years [3]. Invasive listeriosis is reportable in many countries including Canada [1], where the estimated incidence of listeriosis in 2006 was 0.55 cases per 100,000 [4]. Listeriosis can lead to severe outcomes including meningitis, sepsis and death [5, 6]. The severe outcomes are most pronounced in individuals with weakened immune systems, pregnant persons, newborns and elderly people [3]. Pregnant persons and neonates face unique risks, with the potential for foetal transmission and increased risk of pregnancy loss and severe neonatal illness [7].

Cohort studies describing the epidemiology of pregnancy-related listeriosis in France, New Zealand, the United States, the United Kingdom, England and Wales, China, and Israel found a low incidence of pregnancy-related listeriosis, although a significant proportion of cases led to pregnancy loss, neonatal listeriosis, and subsequent neonatal death [8–16]. In Canada, the most recent surveillance study on listeriosis, in 2006, estimated 190 hospitalizations and 44 deaths due to listeriosis, but pregnancy-related cases were not explicitly described [17]. A more recent prospective surveillance study in Canada and Switzerland found the incidence of listeriosis in infants less than six months old to be 1.1 per 100,000 live births per year, but this study did not include pregnant persons [18]. In British Columbia (BC), Canada, the BC Centre for Disease Control (BCCDC) reported seven pregnant cases of listeriosis in the province between 2002 and 2008, four of which resulted in miscarriage or stillbirth [19].

Beyond its health consequences, listeriosis imposes a substantial economic burden due to its high rate of admission to intensive care units and high proportion of fatalities [3, 20]. However, studies estimating the cost burden of listeriosis are few [20–25]. These studies found that despite the incidence of listeriosis being lower than other foodborne pathogens, it ranked as the first or second costliest pathogen per case [22–25]. The annual costs of pregnancy-related listeriosis cases have been reported in the United States, the Netherlands, and South Africa, with the total national economic burden ranging from 379,840 USD to 7,000,000 USD, greatly depending on the types of costs included [22–25]. The cost burden specific to pregnancy-related listeriosis in Canada has not been investigated nor has the relative cost in medical fees for pregnant women and neonates with listeriosis compared to those without listeriosis. Given that listeriosis continues to be a costly issue, this study aimed to describe the epidemiological characteristics and pregnancy outcomes of diagnosed cases of listeriosis, and the excess healthcare costs of pregnant and neonatal cases attributable to listeriosis in BC, Canada, during 2005–2014.

## Methods

### Study design and data

We conducted a retrospective cohort study, as part of a larger project investigating sequelae of 13 pathogens commonly transmitted by food, whose methods are described in detail elsewhere [26, 27]. Briefly, the study population was all residents of BC, Canada, registered in the mandatory provincial health insurance programme during 2005–2014; this programme covers everyone except for First Nations individuals, refugees and recently landed immigrants who have been in the province less than three months, the Canadian military, and the Royal Canadian Mounted Police [28]. The cohort included 5.8 million individuals, followed for an average of 7.5 years/person, including 148 people who each had a single-incident laboratory-confirmed, provincially reported invasive *Listeria* infection, across the 10-year study [27]. For these 148 individuals, listeriosis onset dates were estimated to occur five days before the infection was reported to the province, using median pathogen-specific lag times between onset and report date [27]. In the overall cohort, 219 physician visits (in 63 individuals) and 153 hospitalizations (in 127 individuals) with International Classification of Disease (ICD) codes for listeriosis also occurred during the study [27].

For the present study, we used individually linked data from eight administrative databases that captured all laboratory-confirmed reports of listeriosis [29], and all hospitalizations [30], fee-for-service physician visits, outpatient pharmacy claims, deaths, and stillbirths, as well as demographic and registrations data, over the time period of 2005–2014. Because these data only included variables on sex (not gender), all pregnant persons were coded as female [hereafter called ‘pregnant women’]. Data linkage procedures are described elsewhere [26, 31]. For all databases with ICD codes, we included codes from any diagnostic position.

We defined a case of pregnancy-related listeriosis as a pregnant woman or neonate with either an incident laboratory-confirmed invasive *Listeria* infection (confirmed case) or a physician visit or hospitalization with an ICD code for listeriosis in the absence of laboratory confirmation (likely case). We used the date of physician visit/hospitalization for likely cases as the listeriosis onset date. To ensure we did not miss any cases of listeriosis-related deaths not captured elsewhere, we also searched for ICD codes indicating listeriosis as a cause of death in the death records. To identify whether a case of listeriosis was pregnant, we searched hospitalization data for ICD codes related to birth or other obstetrics, and stillbirths data for the occurrence of a stillbirth (defined as the loss of an infant at or after the 20th week of pregnancy) [32, 33]. We used gestational age recorded in the hospital and stillbirth databases to estimate pregnancy start date, by subtracting it from the date of the hospital admission where the birth, obstetric code or stillbirth was recorded. If an individual’s onset date of listeriosis was between the estimated pregnancy start date and the birth or stillbirth date, we considered them a pregnant case. Neonatal cases were identified using the age (month and year) recorded in the surveillance and demographic databases, and the exact birth date recorded in the hospital database. Those with a listeriosis onset date in 0 to 28 days following their birthdate were considered either early- (0–7 days) or late-onset (8–28 days) neonatal cases. Parent-neonate pairs could not be linked due to data quality issues with the linking variable.

### Ethics and data availability

This study was approved by a University of Waterloo Research Ethics Committee (no 30645), the University of British Columbia Behavioral Research Ethics Board (no H16–00021) and McGill University’s Institutional Review Board (no A03-M12-19A). Access to data provided by the Data Stewards is subject to approval but can be requested for research projects through the Data Stewards or their designated service providers. The following data sets were used in this study: Panorama Public Health Information System, Discharge Abstract Database, Medical Services Plan, PharmaNet, Vital Statistics Deaths, Vital Statistics Stillbirths, Consolidation File and Statistics Canada Income Band Data. Further information regarding these data sets is available on the PopData project webpage at: https://my.popdata.bc.ca/project_listings/15-180/collection_approval_dates.

Per our data access agreement, cell sizes 1–5 cannot be reported to protect privacy, and are either suppressed or presented as ranges (e.g., ‘1–5’) or qualitatively (e.g., ‘very few’).

### Analysis

We described the epidemiology of pregnancy-related listeriosis and conducted a matched cohort study to estimate excess healthcare use and costs in pregnant and neonatal cases attributed to listeriosis. Case characteristics were described using proportions and medians. We estimated the proportion of pregnancies with listeriosis by dividing the number of pregnancies with listeriosis by the total number of deliveries that resulted in a live birth or stillbirth, noting that this denominator does not capture early pregnancy loss or non-hospital deliveries. We estimated the proportion of neonates with listeriosis by dividing the number of neonates with listeriosis by the total number of neonates born in hospital during the study.

Because we could not definitively link parent-neonate pairs, we estimated the incidence in three ways. For the most conservative estimate, we assumed that all neonatal cases were paired to one of the pregnant cases who did not have a stillbirth and used the number of pairs as the numerator. For the least conservative estimate, we assumed none of the pregnant cases and neonatal cases were pairs and used the total number of pregnant plus neonatal cases as the numerator. Finally, for a most likely estimate, we assumed pregnant and neonatal cases who shared a date of delivery/date of birth, local health area (LHA) [34], and neighbourhood income quintile were paired and used the number of pairs plus the number of unpaired pregnant and neonatal cases as the numerator.

We determined case fatality rates by dividing the number of pregnant or neonatal listeriosis cases who died by the number of pregnant or neonatal listeriosis cases. We calculated the proportion of pregnant cases that had a stillbirth, and the median days between estimated pregnancy start, listeriosis onset, and stillbirth. We also calculated the fraction of stillbirths attributable to listeriosis using the population-attributable fraction formula from Mansournia and Altman [35].

To estimate the excess healthcare use and costs among pregnant women and neonates attributable to listeriosis, we used a matched cohort analysis [36]. First, pregnant and neonatal listeriosis cases were matched with pregnant or neonatal-matched comparators without listeriosis at a 1:5 ratio. We selected this ratio because for most cases we were unable to find more than five matches. Individuals were eligible to be matched comparators if they were registered in the provincial health insurance programme for the duration of their pregnancy or neonatal period (defined as 0–28 days of age). For pregnant cases, we exact-matched on comorbidities most relevant to individuals of child-bearing age that could drive healthcare costs during pregnancy (pre-existing diabetes, HIV/AIDS, malignancies, and metastatic tumours); LHA; neighbourhood income quintile; and year. We radius matched on age, categorized into five-year intervals, starting at 15–19 years. The four pre-existing comorbidities were defined using ICD codes for the conditions [33]; individuals were defined as having the comorbidity if they had ICD codes in the hospitalization or physician visit data in two years before their estimated pregnancy start date. For neonatal cases, we exact matched on sex, year, LHA, and neighbourhood income quintile. We radius matched for the month of birth using plus or minus one month of the birth date.

Pregnant cases were followed from their estimated pregnancy start date to two weeks after the date of hospital admission for delivery, to account for those who may have sought care shortly after delivery given that invasive listeriosis treatment typically lasts up to two weeks [37]. Neonates were followed from their birth date to two weeks after their neonatal period (i.e., for 42 days).

In our cost analyses, we included all in-patient hospitalizations, same-day surgeries and procedures, fee-for-service physician visits, and drug dispensing costs for prescription medicines from outpatient pharmacies, which occurred during the follow-up period. We calculated these for pregnant and neonatal cases and their matches as follows. We used case-mix methodology to obtain the costs for each hospital visit [38]. Specifically, we first obtained the resource intensity weight (RIW) for the corresponding case-mix grouping (CMG+) of each hospital visit of interest [38]. We then obtained the provincial-level cost-of-standard-hospital-stay (CSHS) for BC for each year that corresponded to the hospital visits of interest [38] and multiplied this by the RIW. Fee-for-service physician visit costs are based on a fee schedule and are given in the physician record, and prescription drug dispensing costs for all drugs are given in the pharmacy record; these were summed directly.

Analyses were performed in SAS V.9.4 (SAS Institute, Cary, North Carolina, USA). The median and mean number of healthcare visits by type of visit (i.e., in-patient hospital, fee-for-service physician, outpatient pharmacy) and median and mean unadjusted costs per type of visit were calculated for listeriosis cases and matched comparators. Differences in medians were compared using the Mann–Whitney U test due to the non-normal distribution of the data. We used a generalized linear model with a gamma distribution and log link [39] to estimate the cost ratio between total direct healthcare costs of those with listeriosis compared to those without listeriosis, adjusted for age, health authority, neighbourhood income quintile, and gestational diabetes for the pregnant women; and sex, neighbourhood income quintile, and presence of a congenital anomaly for the neonates.

## Results

In BC, Canada, from 2005 to 2014, pregnancy-associated listeriosis reports were rare and accounted for roughly 12% of diagnosed listeriosis cases in the province. Specifically, of the 180 listeriosis cases (148 laboratory-confirmed cases [27], and 32 likely cases identified here), there were 15 (8.3%) pregnant and 7 (3.8%) neonatal cases. No additional cases of listeriosis were identified through listeriosis being a cause of death in the death records. The incidence of pregnancy-related listeriosis ranged from 3.35 (most conservative) to 5.28 (least conservative) per 100,000 pregnancies, with a most likely estimate of 4.56 per 100,000 pregnancies.

All 15 pregnant cases had singleton pregnancies. Listeriosis onset for the pregnant cases occurred in either the second or third trimester, at a median gestational age of 31 weeks (range: 20–39). The median gestational age at delivery was 37 weeks (range: 20–40). At listeriosis onset, the median age of the pregnant cases was 32 years (range: 17–38). All pregnant cases experienced negative outcomes related to their foetus, the most common of which were stillbirth, pre-term birth, and infection of the amniotic sac (each experienced by n = 1–5 cases). Additionally, between 1 and 5 of the 15 pregnant cases experienced gestational diabetes during pregnancy. None experienced listerial sepsis. The vast majority of the 15 cases had an ICD code for listeriosis reported in at least one of their hospital or physician records during the pregnancy. For half the pregnant cases (*n* = 8/15; 53%), the first hospitalization or physician visit for listeriosis was on the same day as the delivery. None of the 15 pregnant cases died during the study period.

There were 2,088 stillbirths reported in BC during 2005–2014, and 1–5 of them occurred among the 15 pregnant listeriosis cases. Among cases who experienced stillbirth, estimated listeriosis onset and stillbirth both occurred at a median of 20 weeks gestation, with a median of 4 days between onset and stillbirth (ranges suppressed). The fraction of stillbirths in the population attributable to diagnosed listeriosis during pregnancy was between 0.045% and 0.237%.

All of the seven neonatal cases were singleton births. Neonatal cases’ median birthweight was 2,710 g (range: 1105–3,206), and while their exact gestational age at birth was unknown, all those with low birth weight (<2,500 g) also had an ICD code for prematurity. These seven neonates experienced sepsis, meningitis, and respiratory distress, and a small fraction had congenital anomalies at birth. Some neonates had early-onset and some had late-onset neonatal listeriosis, and the median number of days between birth and estimated onset date was zero. Most but not all of the seven neonates had an ICD code for listeriosis reported in their hospital or physician records. None of the seven neonates died during the study period.

For our matched cost analysis, the goal of five matches per case was achieved for all pregnant and neonatal cases, with the exception of one pregnant case for whom we found four matches. Additionally, one pregnant case was excluded from the cost analysis due to not having been registered with BC’s medical services plan (MSP) for the entirety of their pregnancy. As with cases, given exact matching, none of the pregnant-matched comparators had pre-existing non-gestational diabetes, tumours, malignancies or HIV/AIDS. Given radius matching for age, the median age of pregnant-matched comparators at the start of follow-up was 31 years (range: 18–37), versus 31.5 years for pregnant cases (range: 16–37). Pregnant-matched comparators had a higher median gestational age at delivery (39 weeks; range: 35–41) than the cases, and a roughly comparable number (16/49; 33%) had gestational diabetes. No stillbirths occurred for pregnant-matched comparators. With exact matching, neonatal matches shared the same sex, LHA, neighbourhood income quintile, and year of birth as the cases. Neonatal-matched comparators had a higher median birthweight (3,404 g; range: 2685–4,380) than cases, and similar to cases, some of the matches had a congenital anomaly at birth.

Pregnant (Tables 1 and 2) and neonatal (Tables 3 and 4) cases with listeriosis had substantially higher healthcare use and costs than their matched counterparts without listeriosis. Both pregnant cases and neonates with listeriosis had on average double the number of unique hospital admissions. The median cumulative days spent in the hospital was about six days longer for pregnant cases with listeriosis (Table 1), and 15 days longer for neonates with listeriosis (Table 3) compared to matched comparators. Neonates with listeriosis also had on average nearly three times as many physician visits during their neonatal period (Table 3); the median number of physician visits for pregnant cases at any time during pregnancy was not significantly different between listeriosis cases and matched comparators (Table 1).

**Table 1.** Healthcare use and costs during pregnancy, from pregnancy start date to two weeks post-birth or stillbirth, for all pregnant cases with *Listeria* and their matched comparators without *Listeria*, in British Columbia, Canada, 2005–2014

	All pregnant cases with listeriosis (n = 14)		Pregnant-matched comparators* without listeriosis (n = 69)	
	Mean (SD)	Median (range)	Mean (SD)	Median (range)
**Healthcare use**				
No. hospital admissions	**1.7 (0.73)**	**2 (1–3)**	**1.1 (0.26)**	**1 (1–2)**
Cumulative days in hospital	**11.2 (7.1)**	**8.5 (2–25)**	**2.6 (2.6)**	**2 (1–15)**
No. fee-for-service physician visits	32.5 (13.0)	31 (11–62)	27.9 (9.91)	26 (6–59)
No. prescription pharmacy claims	**5.7 (7.3)**	**4.0 (0–28)**	**2.2 (3.2)**	**1.0 (0–17)**
**Healthcare costs in Canadian dollars**				
Hospitalizations	**9,530 (5,909)**	**7,826 (2,250–23,889)**	**3,246 (2,037)**	**2,412 (1,660–12,811)**
Fee-for-service physician visits	**4,903 (2,768)**	**4,383 (1,788–10,667)**	**2,440 (852)**	**2,248 (1,299–5,447)**
Prescriptions	180 (230)	75 (0–669)	101 (196)	29 (0–1,053)
Total	**14,613 (6,471)**	**12,633 (4,290– 29,084)**	**5,787 (2,723)**	**4,897 (3,447–18,495)**

* Exact matched on pre-existing diabetes, pre-existing HIV/AIDS, pre-existing malignancies, pre-existing metastatic tumours, local health area, neighbourhood income quintile and year; radius matched on age.

Bolded numbers are statistically significant between groups.

**Table 2.** Association between having listeriosis in pregnancy and total direct healthcare costs during the pregnancy, adjusted for local health area, neighbourhood income quintile, age and gestational diabetes, from the multivariable model

Parameter		Cost ratio	95% CI
Listeria		**2.59**	**(2.07, 3.24)**
Local health area*	1	1.00	(0.45, 2.18)
	2	**0.71**	**(0.52, 0.97)**
	3	**0.72**	**(0.55, 0.93)**
	4	Ref	Ref
Neighbourhood income quintile	1 (lowest)	0.93	(0.71, 1.21)
	2	0.98	(0.73, 1.33)
	3 (highest)	Ref	Ref
Age (years)	15–19	1.10	(0.74, 1.63)
	20–24	0.96	(0.67, 1.34)
	25–29	1.02	(0.82, 1.26)
	30+	Ref	Ref
Gestational diabetes		0.97	(0.84, 1.16)

* There were no cases in health authority 5.

Bolded numbers are statistically significant.

**Table 3.** Healthcare use and costs in the first 42 days of life (i.e., from birth date to two weeks after the 28-day neonatal period), for all neonatal cases with *Listeria* and their matched comparators without *Listeria*, in British Columbia, Canada, 2005–2014

	All neonatal cases with listeriosis (n = 7)	Neonatal-matched comparators* without listeriosis (n = 35)
	Median (range)	Median (range)
**Healthcare use**		
No. hospital admissions	**2 (2–1)**	**1 (1–1)**
Cumulative days in hospital	**17 (11–41)**	**2 (1–77)**
No. fee-for-service physician visits	**11 (2–41)**	**4 (0–13)**
No. prescription pharmacy claims	0 (0)	0 (0–5)
**Healthcare costs in Canadian dollars**		
Hospitalizations	**17,466 (4,718–113,422)**	**835 (716–163,228)**
Fee-for-service physician visits	**2,565 (302–8,812)**	**308 (0–3,838)**
Prescriptions	0 (0)	0 (0–87)
Total	**20,953 (5,952–122,234)**	**1,439 (762–167,067)**

* Exact matched on sex, year, local health area and neighbourhood income quintile; radius matched on age (in months).

Bolded numbers are statistically significant between groups.

**Table 4.** Association between exposure to listeriosis in neonates and total direct healthcare cost during the first 42 days of life, adjusted for sex, income quintile and congenital anomaly, from the multivariable model

Parameter		Cost ratio	95% CI
Listeria		**9.85**	**(5.60, 17.34)**
Sex	Female	1.22	(0.70, 2.12)
	Male	Ref	Ref
Neighbourhood income quintile	1 (lowest)	1.85	(0.86, 3.94)
	2	0.95	(0.47, 1.93)
	3	1.17	(0.45, 3.03)
	5 (highest)	Ref	Ref
Congenital abnormality		**22.53**	**(10.55, 48.12)**

Bolded numbers are statistically significant.

Both pregnant women and neonates with listeriosis had significantly higher median hospital, fee-for-service physician visits, and total costs than their counterparts without listeriosis; median prescription medication costs were significantly higher among pregnant cases but not significantly different among neonatal cases (Table 1, Table 3). The median total healthcare cost for a pregnant case with listeriosis was just over $12,633 Canadian (Table 1). After further adjusting for age, health authority, neighbourhood income quintile, and gestational diabetes, the mean total healthcare cost for those with listeriosis was 2.59 times higher than for pregnant women without listeriosis (Table 2). The median total healthcare cost for neonatal cases with listeriosis was just over $20,953 Canadian (Table 3); means are not presented because of small overall numbers and the presence of congenital abnormalities in some individuals that significantly skewed mean costs. After further adjusting for age, health authority, neighbourhood income quintile, and presence of a congenital anomaly, the mean total healthcare cost for neonates with listeriosis was 9.85 times higher than for neonates without listeriosis (Table 4).

## Discussion

This study assessed, for the first time, the epidemiology and direct healthcare costs of pregnant and neonatal cases of diagnosed invasive listeriosis in a Canadian setting, namely the province of BC during 2005–2014. While the incidence was low and there were no fatalities, complications in pregnancy and during birth were prevalent and excess healthcare costs were high. This study is, to our knowledge, also the first to report the fraction of stillbirths attributable to diagnosed listeriosis in pregnancy, which was between 0.45 and 2.37 per 1,000 stillbirths.

The incidence of pregnancy-related listeriosis in our study was expectedly low, between 3.35 and 5.28 per 100,000 pregnancies. Comparisons of incidence between countries are challenging due to varying case definitions and reporting standards for listeriosis. Nonetheless, our estimate is comparable to that reported in the United States (2.8/100,000 non-Hispanic pregnant women), France (5.6 per 100,000 pregnancies) and the United Kingdom (3.4/100,000 live births) [12, 40, 41].

Here, there were no reported deaths among the listeriosis cases. For pregnant individuals, this was expected, since mortality following listeriosis in pregnancy is rare [9]. Among neonates, case fatality rates range from 0.65% to 21% [8, 9, 10]; given the small number of cases in this study, it is not surprising that there were no infant deaths. Furthermore, while we could not link parents and neonates and therefore did not have data on all neonates of the mothers with listeriosis in this study, we found there were no death records in which listeriosis was listed as the cause of death for infants.

Consistent with other studies [8, 9, 13, 14, 16], here pregnant individuals with listeriosis had infection onset in the second or third trimester. The occurrence of listeriosis later in pregnancy is thought to be due to the suppression of T cells that occurs starting at 26–30 weeks of gestation [42]. However, it is likely that infection in the first trimester leading to spontaneous abortion often goes underdiagnosed [7]. Furthermore, all pregnant cases in this study had experienced negative outcomes affecting their pregnancy and foetuses. It is possible that obstetrical and neonatal complications prompted testing and diagnosis of listeriosis among pregnant women, given the median time between delivery and the first appearance of an ICD code for listeriosis was zero days.

Among the neonatal cases, the most frequently documented birth complications included sepsis, meningitis, and respiratory distress, consistent with previous epidemiological studies [9, 11, 43]. The neonates in our study had a median birthweight of 2,710 g, which is higher than other studies [9, 18]. In BC, listeriosis is usually reported for either the pregnant woman or the neonate, but not both. As we could not link parents and neonates, we lacked birthweight information for any neonates whose infections were unreported, potentially leading to higher birthweight estimates. Furthermore, the neonates in our study who had low birth weight also had an ICD code for prematurity in their hospital records, implying that low birth weight was a secondary outcome to premature birth.

The proportion of pregnant women in this study who experienced a stillbirth was between 6.7 and 33.3%, consistent with other studies that report a foetal loss in 16% to 45% of pregnant listeriosis cases [8–10, 13]. This proportion (6.7% to 33.3%) is exponentially higher than the overall proportion of total births that resulted in a stillbirth in BC between 2005 and 2014 (0.84%) [44].

An epidemiological study in the United States found high rates of hospitalizations among pregnant women and neonates with listeriosis and significantly longer delivery hospital stays for pregnant women with listeriosis compared to those without [11]. Specifically, pregnant women with listeriosis spent on average 4.0 days in hospital during delivery, compared to 2.3 days among pregnant women without listeriosis [11]. Here, we found that pregnant women with listeriosis spent a median of 8.5 cumulative days in hospital during pregnancy compared to 2.0 days among those without listeriosis. We also found that the median length of hospital stay for a neonate with listeriosis was 17 days, similar to findings from the United States (14 nights) and France (16 days) [10, 43].

In terms of direct healthcare costs, both pregnant and neonatal listeriosis cases incurred substantially higher average hospital, physician, and total healthcare costs compared to those without listeriosis. The mean total cost per case for pregnant women ($14,613 CAD during the study period; $19,207 in 2024 CAD) is lower than estimates from the United States (mean of $12,117 in 1993 USD; $36,238 in 2024 CAD) [45]. The mean total cost per neonatal case is also lower than estimates from the same study in the United States. However, again, we have chosen not to report means for neonatal cases here given significant outliers. Lower mean total costs in our study compared to US estimates align with general differences in overall health expenditure per capita [46], but specific reasons related to listeriosis could not be compared. Cost estimates published since 1993 have either only provided total costs, not cost per case, or included indirect or societal costs in their calculations [22–24]. Future estimates that are national in scope and include indirect or societal costs of pregnancy-related listeriosis are needed for Canada.

This study has several limitations. First, we were unable to link pregnant women to their neonates and vice versa, which led us to treat all cases in this study as separate listeriosis events, potentially resulting in the omission of pregnant women with unconfirmed infections whose neonates had confirmed infections and vice versa. Additionally, our small sample size was a limitation to how we could analyze and interpret our data. For example, we could not report yearly trends in listeriosis incidence due to small number restrictions, and all results were highly influenced by each individual case. Listeriosis, like many foodborne infections, is underreported [17], and likely even more so for pregnant women in the first trimester. However, we likely got a much more sensitive capture of listeriosis cases in this population after 20 weeks of gestation, as the health of pregnant persons during this time is carefully monitored.

Overall, while pregnancy-related listeriosis was rare in BC from 2005 to 2014 and no deaths were reported, our study revealed that pregnancy complications and stillbirth among pregnant cases, and low gestational age, respiratory distress, sepsis and meningitis among neonates, were common outcomes for these cases. Moreover, the healthcare costs for pregnancy-related listeriosis cases were substantially higher than in pregnant women and neonates without listeriosis, emphasizing the need to consider these costs when evaluating the cost–benefit of prevention interventions. Increased awareness, education on safe food handling practices, and targeted interventions for pregnant persons could help prevent these cases, ultimately reducing the associated health complications and healthcare costs.
